# Supplementary material for: Protein-altering variants at copy number-variable regions influence diverse human phenotypes
Source: Nat Genet. 2024 Mar 28;56(4):569–78. doi: 10.1038/s41588-024-01684-z (PMC11018521; doi:10.1038/s41588-024-01684-z)
Supplement: Supplementary file 2 — Reporting Summary [file 41588_2024_1684_MOESM2_ESM.pdf]

nature portfolio

Corresponding author(s): Margaux Hujoel, Po-Ru Loh

Last updated by author(s): 1/18/2024

Reporting Summary

Nature Portfolio wishes to improve the reproducibility of the work that we publish. This form provides structure for consistency and transparency in reporting. For further information on Nature Portfolio policies, see our Editorial Policies and the Editorial Policy Checklist.

Statistics

For all statistical analyses, confirm that the following items are present in the figure legend, table legend, main text, or Methods section.

n/a	Confirmed	
☐	☒	The exact sample size (*n*) for each experimental group/condition, given as a discrete number and unit of measurement
☐	☒	A statement on whether measurements were taken from distinct samples or whether the same sample was measured repeatedly
☐	☒	The statistical test(s) used AND whether they are one- or two-sided<br>*Only common tests should be described solely by name; describe more complex techniques in the Methods section.*
☐	☒	A description of all covariates tested
☐	☒	A description of any assumptions or corrections, such as tests of normality and adjustment for multiple comparisons
☐	☒	A full description of the statistical parameters including central tendency (e.g. means) or other basic estimates (e.g. regression coefficient) AND variation (e.g. standard deviation) or associated estimates of uncertainty (e.g. confidence intervals)
☐	☒	For null hypothesis testing, the test statistic (e.g. *F*, *t*, *r*) with confidence intervals, effect sizes, degrees of freedom and *P* value noted<br>*Give P values as exact values whenever suitable.*
☒	☐	For Bayesian analysis, information on the choice of priors and Markov chain Monte Carlo settings
☒	☐	For hierarchical and complex designs, identification of the appropriate level for tests and full reporting of outcomes
☐	☒	Estimates of effect sizes (e.g. Cohen's *d*, Pearson's *r*), indicating how they were calculated

Our web collection on statistics for biologists contains articles on many of the points above.

Software and code

Policy information about availability of computer code

Data collection	None
Data analysis	Custom code used to perform haplotype-informed CNV analysis of UKB WES data has been deposited at Zenodo (10.5281/zenodo.10529671). The following open-source software packages were also used: samtools v1.11, mosdepth v0.2.5, bedtools v2.27.1, BLAT v35, BWA v0.7.17, HTSbox r345, plink v1.9, plink v2.0, BOLT-LMM v2.4.1, REGENIE v2.2.4, SuSiE v0.12.27, R (3.6.3).

For manuscripts utilizing custom algorithms or software that are central to the research but not yet described in published literature, software must be made available to editors and reviewers. We strongly encourage code deposition in a community repository (e.g. GitHub). See the Nature Portfolio guidelines for submitting code & software for further information.

Data

Policy information about availability of data

All manuscripts must include a data availability statement. This statement should provide the following information, where applicable:

- Accession codes, unique identifiers, or web links for publicly available datasets
- A description of any restrictions on data availability
- For clinical datasets or third party data, please ensure that the statement adheres to our policy

Individual-level CNV calls and continuous-valued estimates of relative copy number in UKB will be returned to UK Biobank. Summary statistics for CNV-phenotype association tests are available at https://data.broadinstitute.org/lohlab/UKB_WES_CNV_sumstats/ and have been deposited at Zenodo (10.5281/

zenodo.10529671). Access to the following data resources used in this study is obtained by application: UK Biobank (http://www.ukbiobank.ac.uk/), BioBank Japan (https://biobankjp.org/en/), All of Us (https://allofus.nih.gov/), GTEx (via dbGaP, https://www.ncbi.nlm.nih.gov/gap/, accession phs000424.v8.p2).

Research involving human participants, their data, or biological material

Policy information about studies with human participants or human data. See also policy information about sex, gender (identity/presentation), and sexual orientation and race, ethnicity and racism.

Reporting on sex and gender	We included genetically-determined biological sex as a covariate in analyses.
Reporting on race, ethnicity, or other socially relevant groupings	We used self-reported ethnic background (UK Biobank Data-Field 21000) to define the primary analysis set and to compare allele frequencies across ethnic groupings.
Population characteristics	Prospective cohort study (~500,000 individuals from across the United Kingdom); individuals were between 40 and 69 years old at recruitment (Sudlow et al. 2015 PLOS Medicine).
Recruitment	Recruitment into UK Biobank has been described previously (Sudlow et al. 2015 PLOS Medicine).
Ethics oversight	Ethics approval for the UK Biobank study was obtained from the North West Centre for Research Ethics Committee (Bycroft et al. 2018 Nature). The present study analyzed de-identified data previously collected by UK Biobank and did not require additional ethics oversight.

Note that full information on the approval of the study protocol must also be provided in the manuscript.

Field-specific reporting

Please select the one below that is the best fit for your research. If you are not sure, read the appropriate sections before making your selection.

☒ Life sciences ☐ Behavioural & social sciences ☐ Ecological, evolutionary & environmental sciences

For a reference copy of the document with all sections, see nature.com/documents/nr-reporting-summary-flat.pdf

Life sciences study design

All studies must disclose on these points even when the disclosure is negative.

Sample size	We conducted genetic association analyses on 454,682 individuals (all UK Biobank participants of self-reported White ethnicity who were not excluded by one of the filters below).
Data exclusions	We excluded individuals with trisomy 21, blood cancer, aberrantly many CNV calls, and those who had withdrawn at the time of our study.
Replication	We analyzed the All of Us and BioBank Japan data sets to replicate the key associations identified in UK BIobank; all key associations replicated.
Randomization	Not applicable to our study; participants were analyzed together and not allocated into groups.
Blinding	Not applicable to our study; all data were previously collected, and participants were not allocated into groups.

Reporting for specific materials, systems and methods

We require information from authors about some types of materials, experimental systems and methods used in many studies. Here, indicate whether each material, system or method listed is relevant to your study. If you are not sure if a list item applies to your research, read the appropriate section before selecting a response.

Materials & experimental systems

n/a	Involved in the study
☒	☐ Antibodies
☒	☐ Eukaryotic cell lines
☒	☐ Palaeontology and archaeology
☒	☐ Animals and other organisms
☒	☐ Clinical data
☒	☐ Dual use research of concern
☒	☐ Plants

Methods

n/a	Involved in the study
☒	☐ ChIP-seq
☒	☐ Flow cytometry
☒	☐ MRI-based neuroimaging
